# Influence of Dietary Forage Neutral Detergent Fiber on Ruminal Fermentation, Chewing Activity, Nutrient Digestion, and Ruminal Microbiota of Hu Sheep

**DOI:** 10.3390/ani15030314

**Published:** 2025-01-23

**Authors:** Zhian Zhang, Fei Li, Fadi Li, Zongli Wang, Long Guo, Xiuxiu Weng, Xuchun Sun, Zhenhu He, Xianyu Meng, Zhaoqing Liang, Xiong Li

**Affiliations:** 1 State Key Laboratory of Grassland Agro-Ecosystems, Key Laboratory of Grassland Livestock Industry Innovation, Ministry of Agriculture and Rural Affairs, Engineering Research Center of Grassland Industry, Ministry of Education, College of Pastoral Agriculture Science and Technology, Lanzhou University, Lanzhou 730020, China; 2 Linxia Animal Husbandry Technical Extension Station, Linxia 731199, China; 3 Linxia County Animal Husbandry Development Centre, Linxia 731804, China; 4 Dongxiang County Animal Husbandry Development Centre, Linxia 731199, China; 5 Linxia State Xiaokangcun Feed Co., Linxia 731801, China; 6 Hezheng County Animal Husbandry Development Centre, Linxia 731299, China

**Keywords:** chewing activity, forage neutral detergent fiber, fermentation, ruminal bacterial community, pelleted total mixed ration, sheep

## Abstract

As the key components of dietary carbohydrates, ensuring a balance between forage-neutral detergent fiber (FNDF) and rumen-degradable starch (RDS) is essential for ruminant health. Eight male Hu sheep equipped with rumen cannulas were randomly divided into four groups based on dietary FNDF content: low FNDF (L-FNDF, 6.08%), middle low FNDF (ML-FNDF, 9.47%), middle high FNDF (MH-FNDF, 12.48%), and high FNDF (H-FNDF, 15.68%), while the RDS levels (15.65% of DM on average) were similar among the four groups. A replicated 4 × 4 Latin square design was employed in this study. The results indicated that mean and minimum ruminal pH increased linearly with increasing dietary FNDF content, while the duration and area of pH under 5.8 and 5.6, along with the acidosis index, reduced linearly (*p* ≤ 0.002). There were no differences between the MH-FNDF group and the H-FNDF group in these indicators (*p* > 0.05). The molar proportions of acetate, butyrate, isobutyrate, and isovalerate, as well as the acetate-to-propionate ratio, increased linearly, while propionate and valerate molar proportions and lactate concentration displayed a linear decrease with increasing FNDF content in the diet (*p* < 0.001). Increasing dietary FNDF content extended ruminating and chewing time while enhancing ruminal microbial diversity, promoting the proliferation of Fibrobacterota and *Butyrivibrio* in the rumen, and improving fiber degradability (*p* < 0.05). When the dietary FNDF content exceeded 12.48%, no effects of FNDF on acetate to propionate ratio and fiber utilization were observed (*p* > 0.05). The results suggest that augmenting FNDF content in the PTMR can reshape ruminal fermentation towards acetate production and promote rumination to enhance ruminal pH, thereby alleviating the risk of ruminal acidosis. When the RDS content in the PTMR was 15.57%, an FNDF content of 12.48% was optimal for maintaining stable ruminal function in sheep, and the recommended ratio of FNDF to RDS was 0.8.

## Simple Summary

The role of dietary forage neutral detergent fiber in maintaining rumen function is critical for ruminants, yet it remains unclear what an optimal proportion of forage neutral detergent fiber that is suitable for sheep is in the pelleted total mixed ration feeding model. This investigation aimed to explore the influence of dietary forage neutral detergent fiber on ruminal pH, fermentation characteristics, chewing activity, nutrient digestibility, and the ruminal bacterial community of Hu sheep fed a pelleted total mixed ration. The results showed that increasing dietary forage neutral detergent fiber content shifted the rumen fermentation pattern towards acetate production, reduced lactate concentration in the rumen, and stimulated both chewing and ruminating, thereby mitigating the severity of subacute rumen acidosis in sheep. Additionally, increasing dietary forage neutral detergent fiber content increased the proliferation of cellulolytic bacteria in the rumen, and it promoted fiber utilization. A ratio of forage neutral detergent fiber (12.48%) to rumen-degradable starch (15.57%) with 0.8 was found to be optimal for maintaining stable ruminal function.

## 1. Introduction

In regions with limited access to high-quality forages, such as China, the prevalent dietary strategy for high-yielding ruminants involves feeding an excess of rapidly fermentable carbohydrates to meet energy requirements [1,2]. However, due to deficient physically effective fiber in the diet, this approach leads to an unbalanced diet structure and induces subacute rumen acidosis (SARA) in ruminants, which thereby adversely affects ruminal function and fiber digestion [3]. Subacute ruminal acidosis is generally believed to occur when the duration of ruminal pH under 5.6 is above 3 h/day. Physically effective neutral detergent fiber (peNDF) or forage neutral detergent fiber (FNDF) and rumen degradable starch (RDS) are pivotal dietary constituents for ruminants [4,5]. Maintaining a balanced ratio of peNDF to RDS or FNDF to RDS in the diet can enhance feed efficiency, mitigate SARA risk, uphold optimal ruminal metabolism, and ensure the overall health of ruminants.

Given that the dietary carbohydrate balance predominantly relies on the levels of peNDF or FNDF and RDS, it is essential to evaluate the effects of their respective dietary contributions on rumen health in ruminants when controlling for another variable is consistent. In our earlier study [5], maintaining a constant proportion of FNDF at 9.6% across four experimental groups, we found that increasing the proportion of RDS content (9.70%, 12.00%, 16.00%, 23.66% of DM) in the diets of sheep amplified the risk of SARA, altered fermentation dynamics to favor propionate generation, and diminished the utilization of dietary fibers. In addition, it also indicated that the RDS content of 12.00% was optimal to maintain rumen health when FNDF content was 9.6% [5]. On the contrary, previous studies reported that increasing peNDF content proved advantageous in boosting ruminal pH by enhancing chewing and ruminating to stimulate salivation in lactating dairy cows fed a high-grain diet [1]. Salivary buffering serves as a crucial mechanism for acid neutralization, contributing to about 37% of the total ruminal buffering capacity [6]. In some cases, cattle receiving diets rich in concentrate deliberately opt for longer forage particles to stimulate salivation, as a behavioral adaptation believed to respond to low ruminal pH [7,8]. But it is well established that the application of pelleted total mixed rations (PTMR) in the feeding regimen of fattening ruminants effectively curtails their sorting behavior. This effect arises from the uniform chopping of roughage in PTMR to a similar length and making pellets [9], which restricts the ability of animals to select longer particle sizes to mitigate the reduction in ruminal pH. Consequently, ensuring sufficient FNDF content in the PTMR would avert SARA occurrences and promote stable ruminal function. Nevertheless, data on balancing dietary carbohydrate profile by regulating dietary FNDF content in ruminants under the PTMR feeding mode remains scant; further investigation is required.

Therefore, we hypothesized that increasing the dietary FNDF content would enhance ruminating behavior, improve ruminal fermentation, and subsequently elevate ruminal pH, thereby mitigating the risk of ruminal acidosis in finishing ruminants consuming a PTMR. This investigation was designed to determine the impacts of varying FNDF content in PTMR on ruminal pH, fermentation characteristics, nutrient digestibility, chewing activity, and the ruminal bacterial community in Hu sheep. Furthermore, we sought to identify the optimal FNDF to RDS ratio that is suitable for sheep in the PTMR feeding model.

## 2. Materials and Methods

This experiment was conducted according to established guidelines of the Animal Care Committee of Lanzhou University (approval number: LZU 201803002).

### 2.1. Animals and Experimental Design

The present investigation was a continuation of our earlier study, focusing on the modulation of dietary FNDF in the reduction of ruminal pH in sheep consuming a PTMR characterized by high levels of RDS. A comprehensive description of the study design has previously been delineated [5]. In brief, we selected eight male Hu sheep (body weight of 59.18 ± 4.94 kg, mean ± SD), each equipped with a rumen cannula, and employed a replicated 4 × 4 Latin square design for this investigation. The PTMR for all sheep was featured with similar CP content (average: 13.92% of DM), RDS levels (average: 15.65% of DM), and metabolizable energy (ME) content (average: 10.04 MJ/kg DM). However, the content of dietary FNDF differed among the four treatments, including the low FNDF group (L-FNDF, 6.08%), the middle low FNDF group (ML-FNDF, 9.47%), the middle high FNDF group (MH-FNDF, 12.48%), and the high FNDF group (H-FNDF, 15.68%), which was achieved by varying roughage corn stover proportion (as shown in Table 1). Each experiment period lasted for 31 d, comprising a 5-d washout period (where all sheep received a washout diet to negate carryover effects, as shown in Supplementary Materials “Section S1.1”), a 15-d adaptation period (during which sheep were fed their respective designated diets), and an 11-d sampling period. The sheep were accommodated in individual cages (size: 140 × 75 × 150 cm). Diets were provided twice daily at 08:00 and 18:00, with clean water available ad libitum throughout the trial. The amount of feed offered and orts for each animal were recorded to determine the average daily feed intake (DMI).

### 2.2. Rumen Sampling and Analysis

During each experimental period, rumen digesta (approximately 100 mL) was collected through rumen cannula every two hours from 08:00 to 18:00 on d 21 and d 22 and passed through four layers of cheesecloth for filtration. Five milliliters of filtrate were blended with 1 milliliter of 25% HPO_3_ to prevent volatilization and stored at −20 °C for volatile fatty acid (VFA) measurement, following the method described by Zhang et al. [5]. Simultaneously, another 15 mL of filtrate of ruminal fluid was collected and stored at −20 °C for the lactate determination, which was performed in accordance with the instructions of the lactate assay kit (A019-2-1, Nanjing Jiancheng Bioengineering Institute, Nanjing, China). In addition, 15 mL of filtrate collected at 0 h before morning feeding was preserved at −80 °C for subsequent bacterial DNA extraction.

### 2.3. Ruminal pH and Chewing Activity

Dynamic fluctuations in ruminal pH were monitored in each sheep from day 23 (08:00 h) to d 25 (08:00 h) of each trial period using a ruminal pH monitoring system, as described by Ma et al. [10]. This system captured pH values at 30 s intervals over a span of 48 h. Ruminal pH data were summarized by calculating mean, maximum, and minimum pH, as well as the duration and area with ruminal pH under 5.8 and 5.6. The acidosis index was calculated as the ratio of the area with pH below 5.8 to dry matter (DM) consumption [11]. Concurrently, chewing behavior of individual sheep was continuously monitored for 48 h using video surveillance from day 23 (08:00 h) to d 25 (08:00 h) of each trial period. Through the viewing of the recorded video by a trained person, the time spent on eating and ruminating in 5 min intervals was recorded, assuming that any activity lasted at least 5 min [12,13]. The total chewing time was determined by adding the durations of eating and ruminating, with data reported on a daily (24 h) basis [1].

### 2.4. In Situ Degradability of Alfalfa

The nylon bag technique was used to determine the degradability of DM, NDF, and ADF of alfalfa hay on day 26 of each period. This methodology was used to assess the influence of FNDF content in the diet on fiber degradation. Initially, alfalfa hay samples underwent grinding and were sieved through a 2 mm sieve. Subsequently, three grams of the ground samples were accurately weighed and placed in nylon bags with a pore size of 50 μm, with three replicates of alfalfa hay samples for each sheep. Following sealing, bags were incubated in the ventral sac of the rumen for 24 h. After incubation, all nylon bags were removed, promptly washed six times with cold water to halt fermentation, and dried at 65 °C for 48 h. The degradability of DM, NDF, and ADF was subsequently analyzed following the methodology outlined by Li et al. [14].

### 2.5. Nutrient Digestion

The measurement of nutrient digestibility was performed on 27 to 31 days of each period, during which feed samples were collected daily before morning feeding. Fecal samples were obtained from the rectum of each sheep at 8:00 h, 16:00 h, and 24:00 h daily and were promptly stored at −20 °C. At the end of each period, the collected feed and fecal samples were mixed according to each individual. For each sheep, 150 g of fecal samples were transferred into a brown vial and mixed with 15 mL of 10% H_2_SO_4_ for subsequent determination of CP. Moreover, an extra 200 g of feces were continuously dried at 65 °C for 72 h. Both feed and dried fecal samples were ground and passed through a 1 mm sieve for subsequent analysis. The samples were assessed for DM, organic matter (OM), and CP according to AOAC protocols [15]. Neutral detergent fiber (NDF) and acid detergent fiber (ADF) were determined with the aid of heat-stable α-amylase and sodium sulfite, including residual ash in the measurements. Acid-insoluble ash was assayed using the method described by Allen [16]. The calculation of nutrient digestibility was referred to Zhang et al. [5].

### 2.6. Rumen Microbial DNA Extraction and Sequencing

The total bacterial DNA extraction from the rumen fluid samples was conducted following the YM + SB procedure outlined by Ma et al. [17]. Following the evaluation of the quality and quantity of the DNA, the V3-V4 regions of the 16S rRNA gene were amplified with primers 341F and 806R [18]. The PCR products were purified utilizing the Qiagen Gel Extraction Kit (Qiagen, Hilden, Germany) and sequenced with the Illumina NovaSeq platform. More details are provided in Supplementary Material (Section S1.2).

### 2.7. Statistical Analysis

The results of ruminal pH, chewing activity, fiber degradability, and nutrient digestibility were analyzed using the MIXED program of SAS 9.4 (SAS Institute Inc., Cary, NC, USA). The fixed effects were treatment*_i_* (*i* = 1–4) and period*_j_* (*j* = 1–4), and the random effects were block*_k_* (*k* = 1–2) and sheep*_l_* (*l* = 1–8). In the assessment of ruminal fermentation parameters, sampling times were incorporated as repeated measurements. Microbial composition data were analyzed via a generalized linear model with gamma distribution as the response variable in SPSS 21.0 software (SPSS Inc., Chicago, IL, USA). Detailed procedures were provided in Supplementary material (Section S1.3). The results were presented as means accompanied by their standard errors. Statistical significance was defined as *p* ≤ 0.05, with a trend towards significance indicated for 0.05 < *p* < 0.10.

## 3. Results

### 3.1. Ruminal pH

The area of pH < 5.80 (*p* = 0.096) and the acidosis index (*p* = 0.081) exhibited a quadratic response trend as dietary FNDF content increased (Table 2), while the area of pH < 5.60 showed a quadratic response (*p* = 0.042). No effect of dietary FNDF content on DMI was observed (*p* = 0.992), but the FNDF intake displayed a linear increase with augmenting FNDF content in the diet (*p* < 0.001). The ruminal mean pH (*p* = 0.002) and minimum pH (*p* < 0.001) increased linearly with increasing dietary FNDF content. In contrast, the duration and area of ruminal pH < 5.8 and 5.6, and the acidosis index demonstrated a linear decrease (*p* < 0.05). The ruminal maximum pH remained similar across the four groups (*p* > 0.05).

### 3.2. Volatile Fatty Acids in the Rumen

As shown in Table 3, increasing dietary FNDF content resulted in a quadratic response for the molar ratios of acetate (*p* = 0.015), propionate (*p* = 0.009), valerate (*p* = 0.007), and acetate to propionate ratio (*p* = 0.018), and a trend for a quadratic response for the molar ratio of butyrate (*p* = 0.072) in the rumen. With an increase in dietary FNDF, there was a linear increase in the molar proportions of acetate (*p* < 0.001), isobutyrate (*p* < 0.001), butyrate (*p* < 0.001), and isovalerate (*p* < 0.001), as well as the acetate to propionate ratio (*p* < 0.001) in the rumen. Conversely, a linear decrease was observed for propionate (*p* < 0.001) valerate molar proportions (*p* < 0.001) and lactate concentration (*p* = 0.002). The total volatile fatty acid (TVFA) concentration in the rumen remained unaffected by dietary FNDF content (*p* > 0.05).

### 3.3. Chewing Activity

There was no observed quadratic response in the chewing activity of sheep as the dietary FNDF content increased (*p* > 0.05; Table 4). Nevertheless, a linear increase was noted in the time spent on ruminating per day (*p* = 0.041), along with a trend towards a linear increase in the total chewing time per day (*p* = 0.052). Moreover, both ruminating time (*p* = 0.019) and total chewing time (*p* = 0.027) spent on per kilogram of dry matter intake exhibited a linear increase with the increasing dietary FNDF content. Alterations in dietary FNDF content did not impact the eating time per day or each kilogram of dry matter intake in sheep (*p* > 0.05).

### 3.4. Nutrient Digestibility and Fiber Degradability

Dietary FNDF content had an effect on the apparent digestibility of DM, organic matter (OM), CP, NDF, and ADF (*p* < 0.05; Table 5). However, there was no difference in starch digestibility among the groups (*p* = 0.407). Specifically, the apparent digestibility of DM in the ML-FNDF group was higher than that in the L-FNDF group (*p* = 0.002), but not different from that in the MH-FNDF and H-FNDF groups (*p* > 0.05). The apparent digestibility of CP and OM in the L-FNDF group was lower than that in the MH-FNDF and H-FNDF groups (*p* < 0.05). The apparent digestibility of NDF and ADF in the ML-FNDF group was higher than that in the MH-FNDF and H-FNDF groups (*p* < 0.05). A quadratic response trend in the apparent digestibility of CP emerged with the increasing dietary FNDF content (*p* = 0.061). As the dietary FNDF content increased, the apparent digestibility of DM (*p* = 0.022), OM (*p* = 0.002), and CP (*p* < 0.001) increased linearly, while the apparent digestibility of NDF (*p* = 0.029) and ADF (*p* = 0.028) exhibited a linear decrease. As can be seen in Table 6, dietary FNDF content had an effect on DM degradability of alfalfa hay (*p* = 0.022), with a tendency to affect the degradability of NDF (*p* = 0.099) and ADF (*p* = 0.057). The DM degradability was higher in the MH-FNDF group than in the ML-FNDF and L-FNDF groups (*p* < 0.05), but no distinction was observed when compared to the H-FNDF group (*p* > 0.05). Furthermore, the degradability of DM (*p* = 0.008), NDF (*p* = 0.035), and ADF (*p* = 0.027) increased linearly with increasing dietary FNDF content.

### 3.5. Rumen Bacterial Communities

The ZOTUs (*p* = 0.098), Chao1 (*p* = 0.061), and Ace index (*p* = 0.083) exhibited a quadratic response trend with increasing dietary FNDF content (Table 7). The Shannon index of the ruminal microbial community displayed a linear increase with increasing dietary FNDF content (*p* = 0.028), while the Simpson index displayed a trend towards linear decrease (*p* = 0.056).

**Figure 1:**
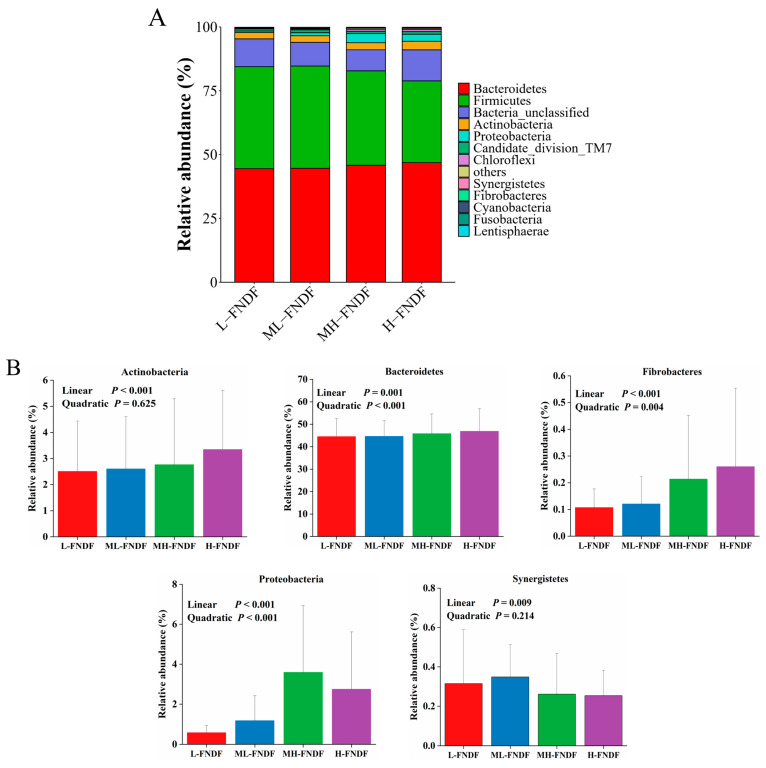
Ruminal microbiota composition at phylum level (**A**) and differential bacteria (**B**) of Hu sheep among the treatments. L-FNDF, low forage neutral detergent fiber; ML-FNDF, middle low forage neutral detergent fiber; MH-FNDF, middle high forage neutral detergent fiber; H-FNDF, high forage neutral detergent fiber.

The predominant phyla present in the rumen ecosystem were identified as Bacteroidetes and Firmicutes (Figure 1A). As shown in Figure 1B, increasing the dietary FNDF content resulted in a quadratic response in the relative abundance of Bacteroidetes (*p* < 0.001), Fibrobacteres (*p* = 0.004), and Proteobacteria (*p* < 0.001). Additionally, the relative abundance of Actinobacteria (*p* < 0.001), Bacteroidetes (*p* = 0.001), Fibrobacteres (*p* < 0.001), and Proteobacteria (*p* < 0.001) in the rumen increased linearly with increasing dietary FNDF content, while the relative abundance of Synergistetes decreased linearly (*p* = 0.009).

At the genus level (Table 8), when dietary FNDF content increased, the relative abundances of Bacteria_unclassified (*p* = 0.027), Erysipelotrichaceae_unclassified (*p* = 0.001), Bacteroides (*p* < 0.001), S24_7_unclassified (*p* < 0.001), Butyrivibrio (*p* = 0.004), and RF16_unclassified (*p* = 0.002) in the rumen had a quadratic response. The relative abundances of Lachnospiraceae_unclassified (*p* = 0.033), Prevotellaceae_unclassified (*p* = 0.037), Coriobacterineae (*p* = 0.026), RF16_unclassified (*p* < 0.001), and Syntrophococcus (*p* = 0.049) in the rumen decreased linearly as the dietary FNDF content increased, while the relative abundances of Prevotella (*p* = 0.072) and Firmicutes_unclassified (*p* = 0.080) tended to decrease linearly. The relative abundances of Incertae_Sedis (*p* < 0.001), Succinivibrio (*p* < 0.001), Erysipelotrichaceae_unclassified (*p* = 0.006), Bacteroides (*p* < 0.001), and Butyrivibrio (*p* < 0.001) increased linearly with increasing FNDF content in the diet.

## 4. Discussion

In the current study, as expected, the FNDF intake exhibited a linear increase, but DMI remained similar across the four groups when the dietary FNDF content increased from 6.08 to 15.68%. According to previous literature, a linear decrease in DMI in goats was observed with increased dietary peNDF_8.0_ concentrations (from 1.9 to 11.7%) [19]. As revealed in a meta-analysis by Ferraretto et al. [20], a decreased DMI was observed when increasing FNDF content in the diet, which was attributed to the filling effect of forage. Caetano et al. [21] found that DMI of cattle fed a high-concentrate diet has a quadratic response with the increasing FNDF content. The lack of DMI difference in this study would be due to the PTMR feeding, which avoided the sorting behavior of sheep [5]. In addition, roughage in the diet was the standard chopped length, which would not result in a serious filling effect of forage. The FNDF and RDS are key nutrients in ruminant diets, with FNDF promoting saliva production and RDS indicating the contribution of grain-based diets to acid accumulation in the rumen [4,5]. In our earlier study, Zhang et al. [5] found that increasing RDS (9.70~23.66% of DM) content in the diet that controlled for the potential confounding effects of FNDF among treatments increased the risk of SARA in sheep. Nevertheless, increasing peNDF concentration could reduce the risk of SARA in dairy cows fed high-concentrate diets [1]. In the current study, increasing FNDF content indeed resulted in increased ruminal mean pH and minimum pH, accompanied by a concomitant decrease in the diurnal duration of pH < 5.8 and 5.6, the area of pH < 5.8 and 5.6, as well as the acidosis index. These findings suggested a reduction in the severity of rumen acidosis with increasing the dietary FNDF content, consistent with a previous study [1]. However, according to the definition where subacute ruminal acidosis (SARA) is identified by ruminal pH dropping below 5.6 for over three hours daily [22], the sheep across all four treatments exhibited signs of SARA based on this criterion in the present study. Notably, a pH < 5.6 sustained for approximately 217 min/d can be regarded as a mild SARA, which does not negatively affect the populations of ruminal fibrolytic bacteria [23]. In this study, the duration of pH < 5.6 in the MH-FNDF and H-FNDF was 278 and 260 min/d, respectively, which could be considered as mild SARA. These results indicated that increasing dietary FNDF content reduced the severity of SARA in sheep; 12.48% of FNDF in the high-RDS (15.65%) diet was the minimum FNDF level allowed to avoid the negative effects of rumen acidosis on ruminal function.

In the present investigation, the increased acetate molar proportion accompanied by a decline in propionate and valerate molar proportions as dietary FNDF content increased was consistent with the findings by Beauchemin and Yang [24], which was attributed to the increased fiber content in the diet. Fiber degradation promotes acetate production in the rumen, while starch degradation favors propionate production, revealing a positive correlation between acetate-to-propionate ratio and FNDF content but a negative correlation with nonstructural carbohydrate content [1,25]. The increased butyrate molar proportion with increasing dietary FNDF content would be attributed to the increased relative abundance of butyrate-producing bacteria, Butyrivibrio, in the rumen [26]. Notably, the concentration of TVFA in the rumen remained similar across the four groups, while the lactate concentration displayed a linear decrease with increasing FNDF content in the diet in this study. The accumulation of organic acids, including volatile fatty acids and lactate, serves as a prominent driver of subacute ruminal acidosis (SARA) in ruminants [27], which suggested that lactate was the main factor to affect the risk of SARA in sheep in the present study.

No difference in the eating time (min/d or min/kg DM) was observed with increasing dietary FNDF content in this study, which was consistent with the observed similar DMI. This finding was consistent with the report by Cao et al. [1], which indicated that ruminating, rather than eating behavior, was more responsive to variations in peNDF_8.0_ concentration in high-concentrate diets. Ruminating and chewing activities are important to regulate the ruminal function of ruminants, which neutralizes approximately 30 to 40% of organic acids produced in the rumen through stimulating saliva secretion [6,16,28]. As expected, increasing FNDF content in the diet led to an increase in the average time spent on ruminating and total chewing per day or per kilogram of DMI, which underscored the mitigation of SARA severity achieved by stimulating salivary flow and finally enhancing ruminal buffering.

Increasing dietary FNDF content would enhance ruminal NDF and ADF digestibility, aligning with the observed increase in the degradability of 24 h in situ NDF and ADF of alfalfa hay in the current study. This phenomenon might be attributed to the higher ruminal pH and the increased substrate that was beneficial for the proliferation of ruminal cellulolytic bacteria with increasing the dietary FNDF content [5,29], which was supported by the increased abundance of Fibrobacterota and Butyrivibrio in the rumen. However, it was interesting that the apparent total-tract digestibility of NDF and ADF exhibited a decline with increasing dietary FNDF content in the present study, while the total-tract starch digestibility remained similar among the four groups. The decreased apparent total-tract digestibility of NDF and ADF was attributed to the increased flow of NDF and ADF to the hindgut (especially the large intestine) with the increase in FNDF content in the diet, where the NDF and ADF digested inefficiently. Increasing dietary FNDF content would not be beneficial for ruminal starch digestibility; however, the lower ruminal starch digestibility with increasing FNDF content in the diet was compensated by higher intestinal starch digestibility [30], thus no differences were observed across the four groups in the current study. Increased digestibility of DM, OM, and CP with increasing FNDF content was due to the increased ruminal pH, which improved the microbial diversity in the rumen, as shown by the increased Shannon index in the present study. In addition, ZOTUs, Chao1, and Ace indexes had a quadratic response, which indicated that an appropriate FNDF content could increase ruminal microbial richness, and 12.48% of FNDF was recommended in the present study.

In the present study, Bacteroidetes and Firmicutes were the predominant bacterial phyla in the rumen, which was consistent with previous reports [5,31,32]. Increasing the FNDF content in the diet increased the relative abundance of Fibrobacterota, Actinobacteria, and Bacteroidetes in the rumen. Bacteroidetes are gram-negative bacteria and sensitive to ruminal pH [33]. The ruminal pH exhibited an increase with increasing the dietary FNDF, which facilitated the proliferation of Bacteroidetes in the rumen. Fibrobacterota and Actinobacteria contribute to the decomposition of plant cellulose [34,35], increasing the FNDF content in the diet provided adequate substrate for these bacteria in the current study. Reduced risk of SARA would increase the relative abundance of Proteobacteria [36]. Increasing FNDF content in the diet reduced the risk of SARA in this study, which promoted Proteobacteria proliferation. Butyrivibrio and Ruminococcus are known to be cellulolytic bacteria, whereas Prevotella is known for the utilization of starch and protein [31,37,38]. In the present study, the relative abundance of Butyrivibrio increased with increasing FNDF content in the diet, while the Ruminococcus increased numerically, which was attributed to the increased nutrient substrates provided by FNDF in the diet. Additionally, the proliferation of cellulolytic bacteria in the rumen requires branched-chain fatty acids (isobutyrate, isovalerate), and the increased molar ratio of isobutyrate and isovalerate in the rumen promoted the multiplication of cellulolytic bacteria in the rumen [39]. Lachnospiraceae_unclassified ferments glucose and fermentable carbohydrates to produce lactate [40]; the decreased relative abundance of this bacteria was consistent with the declined concentration of lactate in the rumen.

## 5. Conclusions

The current study indicated that increasing FNDF content in the PTMR rich in RDS reduced the severity of SARA in sheep and improved the ruminal fermentation, microbial diversity, and fiber degradability. When the rumen-degradable starch content was 15.57% in the PTMR, the FNDF content of 12.48% was optimal to uphold stable ruminal function, which suggested that the ratio of FNDF to RDS with 0.8 was recommended for finishing sheep. Of course, further study is needed to investigate the effects of FNDF and RDS at different levels on rumen microbial function and host gene expression by metagenomics and transcriptomics techniques, which will achieve more accurate nutrition supply.

## Figures and Tables

**Table 1:** Composition and nutrient composition of the experimental diet.

Items	Diet ^1^			
	L-FNDF	ML-FNDF	MH-FNDF	H-FNDF
Corn stover	9.50	14.80	19.50	24.50
Corn bran	24.00	16.00	13.80	5.30
Corn	35.00	37.00	39.00	41.00
Molasses	4.00	4.00	4.00	4.00
Corn germ meal	11.80	11.50	4.10	4.75
Cottonseed meal	7.00	7.00	8.00	8.50
Soybean meal	6.50	7.50	9.40	9.75
Limestone	1.20	1.20	1.20	1.20
NaCl	0.50	0.50	0.50	0.50
Premix ^2^	0.50	0.50	0.50	0.50
Total	100.00	100.00	100.00	100.00
Nutrient content, % of DM				
CP	13.92	13.91	13.89	13.96
Ca	0.79	0.81	0.84	0.86
P	0.28	0.29	0.29	0.31
Starch	25.66	25.66	25.77	25.81
NDF	33.96	32.04	31.13	29.17
ADF	13.94	14.51	15.26	15.79
ME, MJ/kg	10.13	10.08	9.99	9.95
RDS	15.70	15.70	15.57	15.61
FNDF	6.08	9.47	12.48	15.68
FNDF/RDS	0.4	0.6	0.8	1.0

^1^ L-FNDF, low forage neutral detergent fiber; ML-FNDF, middle low forage neutral detergent fiber; MH-FNDF, middle high forage neutral detergent fiber; H-FNDF, high forage neutral detergent fiber; CP, crude protein; NDF, neutral detergent fiber; ADF, acid detergent fiber; RDS, rumen degradable starch; FNDF, forage neutral detergent fiber. ^2^ The premix provided the following per kg of diets: Fe 25 mg; Mn 40 mg; Zn 40 mg; Cu 8 mg; I 0.3 mg; Se 0.2 mg; Co 0.1 mg; VA 1500 IU; VD3 280 IU; VE 20 IU.

**Table 2:** Ruminal pH of Hu sheep among the four groups.

Items ^1^	L-FNDF	ML-FNDF	MH-FNDF	H-FNDF	SEM	*p* Value		
						Treatment	Linear	Quadratic
DMI, kg/d	1.62	1.61	1.63	1.59	0.049	0.992	0.914	0.986
FNDFI, kg/d	0.10 ^d^	0.15 ^c^	0.20 ^b^	0.25 ^a^	0.008	<0.001	<0.001	0.654
Mean pH	5.74 ^c^	5.81 ^bc^	5.93 ^ab^	5.95 ^a^	0.042	0.014	0.002	0.596
Maximum pH	6.89	6.86	6.84	6.85	0.045	0.908	0.552	0.696
Minimum pH	4.86 ^b^	4.96 ^b^	5.18 ^a^	5.13 ^a^	0.046	0.001	<0.001	0.123
Duration pH < 5.8, min/d	815 ^a^	809 ^a^	599 ^ab^	503 ^b^	75.2	0.059	0.013	0.815
Duration pH < 5.6, min/d	645 ^a^	523 ^ab^	278 ^bc^	260 ^c^	70.8	0.009	0.002	0.281
Area pH < 5.8, pH × min/d	371 ^a^	272 ^a^	136 ^b^	133 ^b^	33.8	0.001	<0.001	0.096
Area pH < 5.8, pH × min/d	226.64 ^a^	141.01 ^b^	54.37 ^c^	74.35 ^bc^	23.151	0.001	<0.001	0.042
Acidosis index	235.94 ^a^	167.44 ^b^	84.83 ^c^	97.59 ^c^	21.296	0.001	<0.001	0.081

^1^ L-FNDF, low forage neutral detergent fiber; ML-FNDF, middle low forage neutral detergent fiber; MH-FNDF, middle high forage neutral detergent fiber; H-FNDF, high forage neutral detergent fiber; DMI, dry matter intake; FNDFI, forage neutral detergent fiber intake. ^a–d^ Values within a row with uncommon letters differ among the four groups (*p* < 0.05).

**Table 3:** Ruminal fermentation of Hu sheep among the four groups.

Items ^1^	L-FNDF	ML-FNDF	MH-FNDF	H-FNDF	SEM	*p* Value				
						Treatment	Time	T × T ^2^	Linear	Quadratic
TVFA, mmol/L	68.48	69.94	69.34	72.66	2.158	0.552	<0.001	0.634	0.217	0.667
Acetate, %	51.66 ^c^	54.36 ^b^	57.29 ^a^	56.64 ^a^	0.679	<0.001	<0.001	0.754	<0.001	0.015
Propionate, %	32.71 ^a^	29.19 ^b^	23.39 ^c^	24.40 ^c^	0.864	<0.001	0.001	0.924	<0.001	0.009
Isobutyrate, %	0.63 ^b^	0.70 ^b^	0.68 ^b^	0.85 ^a^	0.031	<0.001	<0.001	0.615	<0.001	0.112
Butyrate, %	11.66 ^c^	12.72 ^b^	14.93 ^a^	14.62 ^a^	0.378	<0.001	0.019	0.747	<0.001	0.072
Isovalerate, %	0.95 ^c^	1.23 ^b^	1.30 ^b^	1.64 ^a^	0.067	<0.001	<0.001	0.945	<0.001	0.721
Valerate, %	2.39 ^a^	1.87 ^b^	1.67 ^b^	1.77 ^b^	0.111	<0.001	0.182	0.900	<0.001	0.007
Acetate: propionate	1.86 ^c^	2.16 ^b^	2.68 ^a^	2.55 ^a^	0.090	<0.001	<0.001	0.750	<0.001	0.018
Lactate, mmol/L	1.47 ^a^	0.86 ^b^	0.76 ^b^	0.65 ^b^	0.15	0.010	-	-	0.002	0.128

^1^ L-FNDF, low forage neutral detergent fiber; ML-FNDF, middle low forage neutral detergent fiber; MH-FNDF, middle high forage neutral detergent fiber; H-FNDF, high forage neutral detergent fiber; TVFA, total volatile fatty acid. ^2^ T×T, the interaction of treatment and time. ^a–c^ Values within a row with uncommon letters differ among the four groups (*p* < 0.05).

**Table 4:** Chewing activity of Hu sheep among the four groups.

Items ^1^	L-FNDF	ML-FNDF	MH-FNDF	H-FNDF	SEM	*p* Value		
						Treatment	Linear	Quadratic
Eating time, min/d	86.56	103.13	96.56	97.19	9.286	0.660	0.554	0.408
Ruminating time, min/d	180	203	234	253	24.5	0.209	0.041	0.942
Total chewing time, min/d	267	306	331	351	28.6	0.244	0.052	0.738
Eating time, min/kg DM	54.90	67.41	60.46	62.64	6.300	0.582	0.574	0.428
Ruminating time, min/kg DM	109	127	141	161	14.0	0.116	0.019	0.980
Total chewing time, min/kg DM	164	195	202	223	16.5	0.138	0.027	0.776

^1^ L-FNDF, low forage neutral detergent fiber; ML-FNDF, middle low forage neutral detergent fiber; MH-FNDF, middle high forage neutral detergent fiber; H-FNDF, high forage neutral detergent fiber.

**Table 5:** Nutrient apparent digestibility of Hu sheep among the four groups.

Digestibility ^1^	L-FNDF	ML-FNDF	MH-FNDF	H-FNDF	SEM	*p* Value		
						Treatment	Linear	Quadratic
DM, %	71.59 ^b^	73.40 ^a^	73.22 ^ab^	73.12 ^ab^	0.712	0.002	0.022	0.535
OM, %	73.46 ^b^	75.46 ^a^	75.52 ^a^	75.81 ^a^	0.681	0.001	0.002	0.403
CP, %	80.56 ^b^	81.64 ^ab^	81.99 ^a^	82.67 ^a^	0.548	0.001	<0.001	0.061
NDF, %	52.07 ^ab^	54.59 ^a^	50.00 ^b^	49.23 ^b^	1.444	0.000	0.029	0.742
ADF, %	47.38 ^ab^	49.73 ^a^	46.22 ^b^	45.08 ^b^	1.289	0.000	0.028	0.398
Starch, %	98.63	98.74	98.64	98.65	0.051	0.407	0.904	0.319

^1^ L-FNDF, low forage neutral detergent fiber; ML-FNDF, middle low forage neutral detergent fiber; MH-FNDF, middle high forage neutral detergent fiber; H-FNDF, high forage neutral detergent fiber; DM, dry matter; OM, organic matter; CP, crude protein; NDF, neutral detergent fiber; ADF, acid detergent fiber. ^a,b^ Values within a row with uncommon letters differ among the four groups (*p* < 0.05).

**Table 6:** The 24 h in situ degradability of alfalfa hay.

Degradability ^1^	L-FNDF	ML-FNDF	MH-FNDF	H-FNDF	SEM	*p* Value		
						Treatment	Linear	Quadratic
DM, %	50.16 ^c^	52.25 ^bc^	56.30 ^a^	54.67 ^ab^	1.243	0.022	0.008	0.160
NDF, %	45.65	45.23	47.91	47.84	0.949	0.099	0.035	0.858
ADF, %	38.82	37.88	41.18	41.26	1.034	0.057	0.027	0.621

^1^ L-FNDF, low forage neutral detergent fiber; ML-FNDF, middle low forage neutral detergent fiber; MH-FNDF, middle high forage neutral detergent fiber; H-FNDF, high forage neutral detergent fiber; DM, dry matter; NDF, neutral detergent fiber; ADF, acid detergent fiber. ^a–c^ Values within a row with uncommon letters differ among the four groups (*p* < 0.05).

**Table 7:** Microbial alpha-diversity in rumen of Hu sheep among the four groups.

Items ^1^	L-FNDF	ML-FNDF	MH-FNDF	H-FNDF	SEM	*p* Value		
						Treatment	Linear	Quadratic
ZOTUs	2143	2388	2384	2331	82.9	0.178	0.156	0.098
Chao1	2727	2970	2968	2872	82.2	0.176	0.261	0.061
Ace	2730	2967	2958	2889	80.9	0.193	0.217	0.083
Shannon	5.22	5.36	5.60	5.62	0.128	0.136	0.028	0.639
Simpson	0.03	0.03	0.02	0.02	0.004	0.237	0.056	0.923

^1^ L-FNDF, low forage neutral detergent fiber; ML-FNDF, middle low forage neutral detergent fiber; MH-FNDF, middle high forage neutral detergent fiber; H-FNDF, high forage neutral detergent fiber.

**Table 8:** The relative abundance (top 25) of ruminal microbiota at genus level of Hu sheep among the four groups.

Items ^1^	L-FNDF	ML-FNDF	MH-FNDF	H- FNDF	SEM	*p* Value	
						Linear	Quadratic
*Prevotella*	25.97	25.55	21.39	22.56	1.761	0.072	0.569
Lachnospiraceae_unclassified	12.68	15.19	11.74	9.85	1.208	0.033	0.156
Bacteria_unclassified	10.90	9.27	8.25	12.15	1.263	0.548	0.027
Ruminococcaceae_unclassified	5.98	5.72	6.24	6.17	0.402	0.521	0.751
Prevotellaceae_unclassified	8.20	7.79	6.11	6.69	0.720	0.037	0.146
uncultured	5.71	7.09	7.07	6.67	0.496	0.519	0.051
RC9	4.75	4.96	5.33	5.69	0.489	0.284	0.740
Bacteroidales_unclassified	3.69	2.77	4.21	3.95	0.320	0.144	0.287
Bacteroidetes_unclassified	3.62	3.16	3.12	1.94	0.365	0.320	0.897
Clostridiales_unclassified	2.51	1.96	3.04	2.07	0.180	0.954	0.290
BS11_unclassified	2.22	1.63	2.81	2.67	0.344	0.656	0.444
Incertae_Sedis	1.23	1.01	1.97	1.59	0.115	<0.001	0.694
*Ruminococcus*	1.20	1.79	1.99	2.25	0.249	0.473	0.215
*Coriobacterineae*	1.35	0.89	1.30	0.82	0.121	0.026	0.692
*Bifidobacteriaceae*	0.96	1.16	0.68	1.40	0.159	0.367	0.094
*Succinivibrio*	0.41	0.55	2.03	1.07	0.118	<0.001	0.057
Erysipelotrichaceae_unclassified	0.36	0.71	2.44	0.63	0.061	0.006	0.001
Candidate_division_TM7_unclassified	0.85	0.77	0.80	1.05	0.120	0.220	0.103
Firmicutes_unclassified	0.97	0.95	0.89	0.73	0.101	0.080	0.615
*Bacteroides*	0.55	0.60	0.88	0.76	0.105	<0.001	<0.001
S24_7_unclassified	0.65	0.47	0.76	0.52	0.138	<0.001	<0.001
*Butyrivibrio*	0.41	0.41	0.43	0.65	0.042	<0.001	0.004
RF16_unclassified	0.62	0.33	0.27	0.40	0.044	<0.001	0.002
*Syntrophococcus*	0.67	0.28	0.24	0.27	0.056	0.049	0.204
*Blautia*	0.28	0.32	0.39	0.29	0.032	0.959	0.629

^1^ L-FNDF, low forage neutral detergent fiber; ML-FNDF, middle low forage neutral detergent fiber; MH-FNDF, middle high forage neutral detergent fiber; H-FNDF, high forage neutral detergent fiber.

## Data Availability

All raw sequences were deposited in the NCBI Sequence Read Archive (SRA) database and can be accessed via accession number PRJNA1145563.

## Supplementary Materials

The following supporting information can be downloaded at: https://www.mdpi.com/article/10.3390/ani15030314/s1, Section S1.1: Composition and nutrient composition of the experimental diet during the transition period; Section S1.2: DNA extraction and 16S rRNA sequencing; Section S1.3: Statistical analysis.

## Abbreviations

The following abbreviations are used in this manuscript:

ADF	acid detergent fiber
CP	crude protein
DMI	dry matter intake
FNDF	forage neutral detergent fiber
ME	metabolic energy
NDF	neutral detergent fiber
OM	organic matter
peNDF	physically effective neutral detergent fiber
PTMR	pelleted total mixed ration
RDS	rumen degradable starch
SARA	subacute ruminal acidosis
VFA	volatile fatty acid
