# Supplementary material for: Influence of Dietary Forage Neutral Detergent Fiber on Ruminal Fermentation, Chewing Activity, Nutrient Digestion, and Ruminal Microbiota of Hu Sheep
Source: Animals (Basel). 2025 Jan 23;15(3):314. doi: 10.3390/ani15030314 (PMC11815921; doi:10.3390/ani15030314)
Supplement: Supplementary file 1 [file animals-15-00314-s001.zip › animals-3434919-supplementary.pdf]

section S1.1 Composition and nutrient composition of the experimental diet during the transition period

Table S1. Composition and nutrient composition of the experimental diet during the transition period.

Item	Diet
Ingredient, % of DM	
Corn stover	20.00
Corn bran	28.00
Corn	10.00
Corn gluten meal	30.80
Cottonseed meal	5.00
Molasses	4.00
Limestone	1.20
NaCl	0.50
Premix[1]	0.50
Total	100.00
Nutrient content, % of DM	
DM	91.82
CP	12.95
Starch	13.25
NDF	47.76
ADF	19.36
ME, MJ/kg	9.34
RDS	8.75
FNDF	12.80
FNDF/RDS	1.46

[1]The premix provided the following per kg of diets: Fe 25 mg, Mn 40 mg, Zn 40 mg, Cu 8 mg, I 0.3 mg, Se 0.2 mg, Co 0.1 mg, VA 20,000 IU, VD_3 4,500 IU, VE 20 IU.

section S1.2 DNA extraction and 16S rRNA sequencing

Bacterial DNA was extracted from the rumen fluid samples using the YM+SB method [17]. Amplification of the V3-V4 region of the 16S rRNA gene was performed using primers 341 F (forward, 5'-CCTAYGGGRBGCASCAG-3') and 806 R (reverse, 5'-GGACTACNNGGGTATCTAAT-3') [18]. Polymerase chain reaction (PCR) process entailed the use of 15 μL Phusion® High-Fidelity PCR Master Mix (New England Biolabs), 3 μL primers, 10 μL template DNA, and 2 μL ddH2O. The thermal cycling procedure consisted of an initial denaturation at 98°C for 1 min, followed by 30 cycles of denaturation at 98°C for 10 s, annealing at 50°C for 30 s, extension at 72°C for 30 s, and finally extension at 72°C for 5 min. For quantification and identification of PCR products, an equal volume of IX loading buffer

(containing SYB green) was mixed with PCR products and detected by electrophoresis on a 2% agarose gel. The mixed PCR products were then purified using the Qiagen Gel Extraction Kit (Qiagen, Germany). Sequencing libraries were generated using Illumina TruSeq DNA PCR-Free Library Preparation Kit (Illumina, USA). Library quality was assessed using a Qubit@ 2.0 fluorometer (Thermo Scientific) and an Agilent Bioanalyzer 2100 system [18]. Finally, eligible libraries underwent sequencing on the Illumina NovaSeq platform (Beijing Novozymes Bioinformatics Co., Ltd.), generating 250 bp paired-end reads.

Quality control and sequence pair merging were conducted in accordance with the methodology described by Ma et al. [17]. Subsequently, zero-radius operational classification units (ZOTUs) were clustered using usearch v11. A ZOTU table was generated by mapping length-filtered sequences to representative ZOTU sequences containing amplicon counts for each ZOTU. Classification annotation was performed using Silva.nr.138. Downstream analyses were performed using MiSeq SOP2 (https://mothur.org/wiki/miseq-sop) compliant Mothur v 1.41.1. The richness and diversity of ZOTUs were estimated from observed ZOTUs and faith phylogenetic diversity indices, respectively.

section S1.3 Statistical Analysis

The results of ruminal pH, chewing activity, fiber degradability and nutrient digestibility were analyzed using the MIXED procedure of SAS 9.4 (SAS Institute Inc., Cary, NC, USA) according to the statistical model: $Y_{ijkl} = \mu + treatment_i + period_j + block_k + sheep_l + treatment_i \times period_j + error_{ijkl}$, where Y_{ijkl} is the dependent variable, μ is the overall mean, $error_{ijkl}$ is the residual error. The fixed effects were $treatment_i$ (i = 1-4) and $period_j$ (j = 1-4), and the random effects were $block_k$ (k = 1-2) and $sheep_l$ (l = 1-8). For ruminal fermentation analysis, the sampling time was added in the model previously described and set as repeated measurement. For the analysis of microbial compositional data, the generalized linear model (GLM) with the gamma distribution in SPSS 21.0 software (SPSS Inc., Chicago, IL, USA) was opted for the response variable. The data were presented as means with standard errors. A significance level of $P < 0.05$ was employed, and trends were considered if $0.05 \leq P < 0.10$.
